# Probing the Water Uptake and Phase State of Individual Sucrose Nanoparticles Using Atomic Force Microscopy

**DOI:** 10.1021/acsearthspacechem.1c00101

**Published:** 2021-09-10

**Authors:** Chamika K. Madawala, Hansol D. Lee, Chathuri P. Kaluarachchi, Alexei V. Tivanski

**Affiliations:** Department of Chemistry, University of Iowa, Iowa City, Iowa 52242, United States

**Keywords:** atomic force microscopy, phase state, sucrose, aerosol particles, relative humidity, nanoparticles

## Abstract

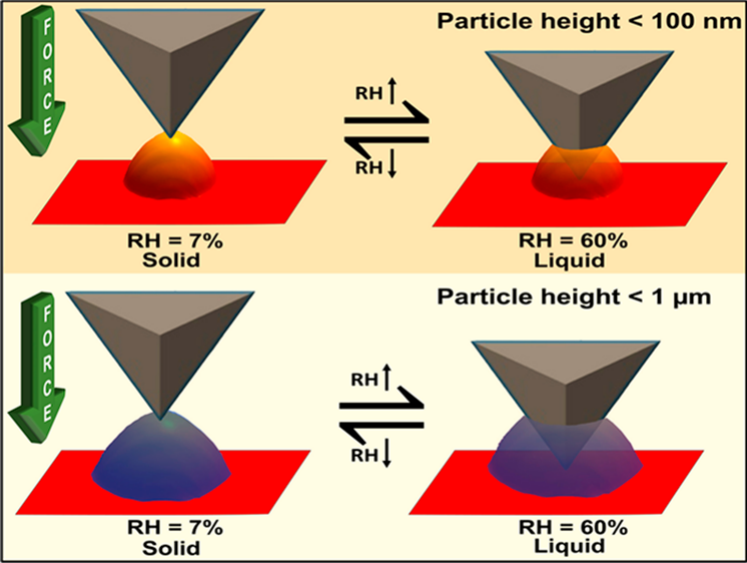

The effects of atmospheric aerosols on the climate and atmosphere of Earth can vary significantly depending upon their properties, including size, morphology, and phase state, all of which are influenced by varying relative humidity (RH) in the atmosphere. A significant fraction of atmospheric aerosols is below 100 nm in size. However, as a result of size limitations of conventional experimental techniques, how the particle-to-particle variability of the phase state of aerosols influences atmospheric processes is poorly understood. To address this issue, the atomic force microscopy (AFM) methodology that was previously established for sub-micrometer aerosols is extended to measure the water uptake and identify the phase state of individual sucrose nanoparticles. Quantified growth factors (GFs) of individual sucrose nanoparticles up to 60% RH were lower than expected values observed on the sub-micrometer sucrose particles. The effect could be attributed to the semisolid sucrose nanoparticle restructuring on a substrate. At RH > 60%, sucrose nanoparticles are liquid and GFs overlap well with the sub-micrometer particles and theoretical predictions. This suggests that quantification of GFs of nanoparticles may be inaccurate for the RH range where particles are semisolid but becomes accurate at elevated RH where particles are liquid. Despite this, however, the identified phase states of the nanoparticles were comparable to their sub-micrometer counterparts. The identified phase transitions between solid and semisolid and between semisolid and liquid for sucrose were at ∼18 and 60% RH, which are equivalent to viscosities of 10^11.2^ and 10^2.5^ Pa s, respectively. This work demonstrates that measurements of the phase state using AFM are applicable to nanosized particles, even when the substrate alters the shape of semisolid nanoparticles and alters the GF.

## Introduction

Exploring the physical–chemical properties of atmospheric aerosols is important because they play a major role in regulating climate-relevant processes.^1−7^ Aerosols can have direct and indirect effects on the climate, leading to radiative forcing.^6^ The direct aerosol effect refers to the ability to scatter and absorb solar radiation, while the indirect effect refers to the aerosols acting as cloud condensation nuclei (CCN) or ice nucleating particles (INPs), facilitating cloud formation.^6,8−11^ A variety of aerosols originate from primary and secondary sources.^12^ The natural and anthropogenic sources give rise to primary aerosols, including soot, volcanic ash, and sea spray aerosols (SSAs).^12,13^ SSAs in particular consist of a highly diverse size-dependent mixture of various organic, inorganic, and biological compounds, including but not limited to salts, saccharides, fatty acids, amino acids, carboxylic acids, and biological debris.^4,14−19^ SSAs are typically super-micrometer (size > 1 μm), sub-micrometer (size < 1 μm), and sub-100 nm in size.^3,17,20^ Secondary aerosols are predominantly generated by oxidation of volatile compounds, followed by condensation of oxidized products, with secondary organic aerosols (SOAs) and secondary marine aerosols (SMAs) as the two common examples.^21−27^ SOAs contain organic compounds, such as organosulfates and carboxylic acids,^27−30^ while SMAs contain sulfates, ammonium, and other organic species.^31^ SOAs and SMAs are typically sub-100 nm in size.^27,32−34^ Collectively, SSAs, SOAs, and SMAs account for a significant fraction of the total mass of atmospheric aerosols.^35,36^

Characterization of sub-100 nm aerosol properties is challenging as a result of their size. First, the small sizes pose significant constraints on existing conventional instrumentation. For example, the bead mobility, poke flow, and optical tweezer techniques are often used to quantify the viscosity to solve for the diffusion constants.^7,37^ However, such measurements are limited to the super-micrometer size range.^21^ Other techniques also exist that can identify the phase state of sub-100 nm particles without measuring the viscosity, such as the particle rebound method.^7,37^ However, the method is only applicable over a relatively narrow range of viscosities. Second, atmospheric aerosols can exhibit size-dependent properties. For example, Hasenecz et al. observed an increase in the organic mass fraction with a decreasing particle size, reaching ∼70% for sub-180 nm SSAs.^38^ Furthermore, the morphologies of SSAs have been found to vary significantly with the particle size.^39−41^ Finally, atmospheric aerosols from the same source and similar size range can exhibit significant particle-to-particle variability.^41^ This requires studies that can be performed on a single particle based on aerosol properties, such as the water uptake and phase state.^41^

The water uptake and phase states of aerosols are important to understand, because they influence the reactivity of aerosols with various atmospheric gases,^42^ SOA formation and partitioning,^43−45^ CCN and water uptake behavior,^8,32,46,47^ heterogeneous and multiphase reactions,^48,49^ and the ability to act as INPs.^50−53^ The size-dependent aerosol composition results in highly variable and relative humidity (RH)-dependent water uptake, which, in turn, affects the phase state by changing the aerosol solute concentration and viscosity.^54^ This is particularly true for sub-100 nm aerosols that are predominantly organic^39^ and, thus, generally have lower water uptake.^17,33,55^

The effects of the aerosol size on the water uptake were reported previously on the basis of the hygroscopic growth factor (GF) measurements.^56^ The GF at a particular RH is defined as the ratio of the aerosol volume-equivalent diameter at a corresponding RH over the dry diameter (ca. 7% RH). As RH increases, aerosols can take up varying amounts of water that usually increases the GF with larger values typically indicative of a more hygroscopic aerosol.^57^ Previously, Biskos et al. demonstrated the effect of the nanoparticle size on water uptake using a humidified tandem differential mobility analyzer (HTDMA), where the size effects can be described by the Kelvin effect. Specifically, a lower GF at 80% RH was observed for deliquesced NaCl nanoparticles (size range of 6–40 nm) compared to their micrometer-sized counterparts.^58−60^ Furthermore, for non-deliquesced NaCl nanoparticles, HTDMA data sometimes revealed a decreasing GF trend with increasing RH ranging between 10 and 70%.^61^ Concurrently, the authors noted a significant change in the nanoparticle shape using transmission electron microscopy, which partially accounted for the observed GF trend.^61−63^ These studies underscore the fact that sub-100 nm aerosols with high surface/volume ratios can display water uptake properties that can be different relative to their larger counterparts (e.g., super- and sub-micrometer sizes). Thus, a simple extrapolation of the properties of larger aerosols onto sub-100 nm sized aerosols can sometimes lead to inaccurate results. Instead, single-particle methods that enable direct measurements of water uptake and identification of the phase state as a function of RH on individual sub-100 nm atmospheric aerosols (e.g., SOAs and SMAs) are required. The phase state measurements over a wide range of sizes may potentially yield to the development of models that could be used to more accurately extrapolate aerosol properties measured on larger aerosols toward smaller sizes.

We previously reported a new method that permits accurate determination of the water uptake and phase state of individual substrate-deposited sub-micrometer aerosols as a function of RH using atomic force microscopy (AFM) imaging and force spectroscopy.^57,64,65^ By varying RH, solid, semisolid, and liquid phase states were directly probed for these sub-micrometer aerosols. For sucrose sub-micrometer particles, the phase measurements showed that the solid to semisolid phase transition occurs at ∼18% RH (corresponding viscosity of 10^11.2^ Pa s), while the semisolid to liquid transition occurs at ∼60% RH (corresponding viscosity of 10^2.5^ Pa s). However, the method was not applied to individual sub-100 nm aerosols. In addition, the AFM method requires a substrate, and the presence of the substrate in some cases may influence measured properties of substrate-deposited particles (e.g., particle shape changes because of the impaction/recovery on a solid substrate).^37^ However, AFM can analyze the data on an individual particle basis, which can potentially reveal important outliers to the aerosol population data that may otherwise go undetected if probed by an ensemble-averaged technique, such as HTDMA.

Here, we extend our previously established AFM methodology to individual sucrose nanoparticles with varying heights below 100 nm. The sucrose nanoparticles were selected as a model system due to two reasons. First, the parametrized relationship between the viscosity, phase state, and RH for sucrose particles is already established,^57,66^ enabling direct comparison between the sub-100 nm and sub-micrometer particles. Second, sucrose shares some functional groups similar to those found in SOAs, and saccharides constitute a significant portion of the organic content in SSAs.^57,64^ In this study, the RH was increased from ∼7 to 80% to measure the GF of several individual sucrose nanoparticles with heights ranging between 50 and 110 nm (volume equivalent diameter range of 100–230 nm). A decreasing trend in the GF was observed with increasing RH up to 60%, which could be attributed to semisolid sucrose nanoparticles restructuring on a solid surface. However, the GF measurements at RH > 60%, where sucrose nanoparticles are liquid, converge with the response quantified on larger particles and overlaps with the theoretical predictions. By employing contact mode AFM force spectroscopy, the solid, semisolid, and liquid phase states of individual sucrose nanoparticles were identified as a function of RH, extending the previously established AFM methodology from sub-micrometer to now include sub-100 nm particle sizes.

## Materials and Methods

### Sucrose Nanoparticle Generation

Sucrose was purchased from Sigma-Aldrich (reagent grade, 99.99% purity) and used without additional purification. A 0.1 M sucrose aqueous solution was atomized with a constant output atomizer (model 3076, TSI, Inc.). The aerosols were substrate-deposited by impaction onto hydrophobically coated silicon wafers using a micro-orifice uniform deposit impactor (MOUDI, model 110, MSP, Inc.).^39,57,64,67^ The silicon wafer was placed on the MOUDI stage 9, which corresponds to the aerodynamic diameter 50% cutoff range of 92–180 nm. Before deposition onto a silicon wafer,^67^ the aerosol stream was mixed with wet air at a constant rate of 20 L/min to achieve ∼80% RH in the mixing chamber.^67^ The substrate-deposited sucrose nanoparticles were stored in clean Petri dishes and kept inside a laminar flow hood (NU-425-400, NuAire, Inc.) at room temperature (20–25 °C) and ambient pressure at 20–25% RH, and all AFM experiments were conducted on the following day.^68^

### AFM Water Uptake and Phase State Measurements

All AFM studies were conducted using a molecular force probe three-dimensional (3D) AFM (Asylum Research, Santa Barbara, CA, U.S.A.). AFM imaging and force measurements were performed at room temperature (20–25 °C) and pressure using silicon nitride probes (model CSC37, MikroMasch) with a nominal spring constant of 1.0 N/m and a typical tip radius of curvature of 10 nm with a scan rate of 1 Hz. The actual AFM cantilever spring constant was determined using the thermal noise method.^69^ AFM height images of individual sucrose nanoparticles were collected at a particular RH using an intermittent contact mode (AC mode). A custom-made humidity cell was used to control the RH with a range between ∼7 and 80%, as described previously.^70^ After each change in RH, 10–15 min of equilibration time was allocated to ensure that the nanoparticles are in thermodynamic equilibrium with the surrounding water vapor.^70^ At a particular RH, the height, projected area diameter, and volume equivalent diameter of individual sucrose nanoparticles were determined from AFM height images.^57,64,71,72^ AFM force spectroscopy studies were performed in contact mode with the maximum applied force of 20 nN. A total of 17 individual sucrose nanoparticles with heights ranging from 50 to 110 nm (volume equivalent diameter range of 100–230 nm) were studied for the water uptake and phase state measurements. For each nanoparticle, five repeated force versus tip–sample separation measurements (i.e., force plots) were collected at an approximate particle center at a particular RH. On the basis of the force plots, the viscoelastic response distance (VRD) and relative indentation depth (RID) values were determined for each nanoparticle at a particular RH, as described previously, with each value reported as an average and one standard deviation.^41,57^

## Results and Discussion

**Figure 1:**
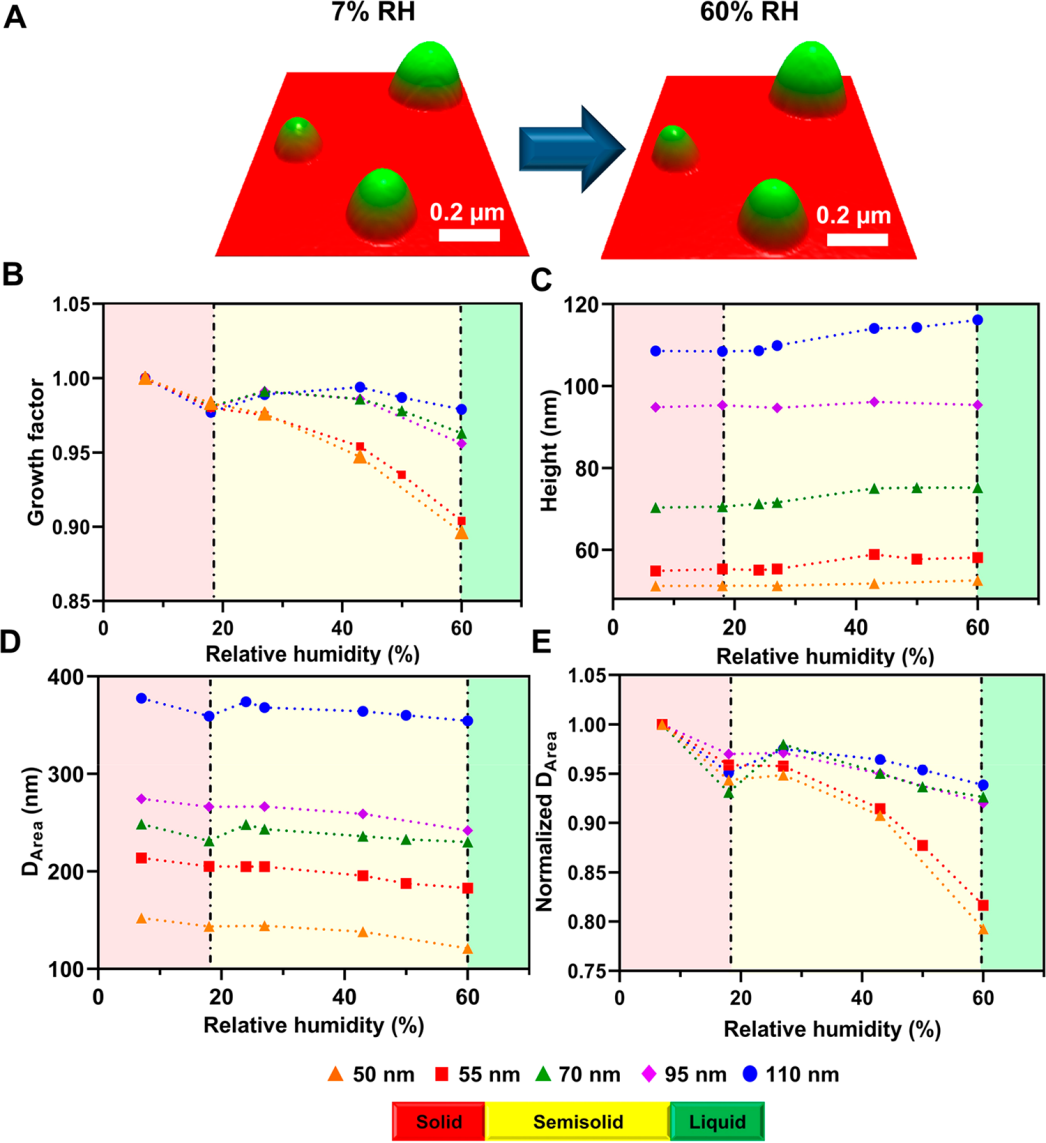
(A) AFM 3D height images of three representative individual sucrose nanoparticles with heights (at 7% RH) of 55, 70, and 110 nm and *D*_vol_ of 130, 155, and 230 nm, respectively, at (left) 7% and (right) 60% RH. Plot of the (B) growth factor, (C) particle height, (D) projected area diameter *D*_area_, and (E) normalized *D*_area_ (relative to 7% RH) versus RH for five selected particles with heights (at 7% RH) of 50 nm (orange), 55 nm (red), 70 nm (green), 95 nm (purple), and 110 nm (blue) with *D*_vol_ of 100, 130, 155, 185, and 230 nm, respectively. The RH range for the solid, semisolid, and liquid phase states is indicated by red, yellow, and green color bars, respectively. The solid to semisolid and semisolid to liquid phase transitions are expected to occur at ∼18 and 60% RH for sucrose, respectively. This figure was reproduced from ref (57). Copyright 2017 American Chemical Society (ACS).

Figure 1A shows AFM 3D height images at 7 and 60% RH of three selected representative individual sucrose nanoparticles with heights (at 7% RH) of 55, 70, and 110 nm (volume equivalent diameter, *D*_vol_, of 130, 155, and 230 nm, respectively). The nanoparticles display rounded morphology consistent with the previous studies on sub-micrometer sucrose particles.^57^ For water uptake, the 3D GF was quantified over each individual nanoparticle at a particular RH value ranging from 7 to 60%, which is defined as the ratio of *D*_vol_ at the corresponding RH over that at 7% RH (eq 1).
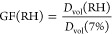
1The GF was decreasing with an increase in RH (Figure 1B). At 60% RH, the GF ranged from 0.89 to 0.98, with smaller nanoparticles displaying lower GF values. We previously reported the GF value of 1.08 at 60% RH for a significantly larger (160 nm particle height and *D*_vol_ of 400 nm at 7% RH) sucrose particle.^57^ Each nanoparticle displayed a modest increase in the particle height as RH increases (Figure 1C) and a concurrent decrease in the projected area diameter *D*_area_ (Figure 1D). Hence, the overall decrease in the GF with increasing RH stems from a significant decrease in the projected particle area that counteracts the increase in height. To ensure that the GF reduction is not due to an imaging artifact as a result of repeated AFM imaging, the experiments were conducted on several different sucrose samples and GF values were also measured during both increasing and decreasing RH (i.e., hydration and dehydration modes), with all measurements yielding similar GF results.

The GF reduction observed here for substrate-deposited sucrose nanoparticles likely originates from the contribution of the solid substrate, which induces the nanoparticle restructuring as RH was increasing (here, the restructuring refers to an increase in the particle height and concomitant *D*_area_ reduction), as also reported previously.^62,63,73,74^ Assuming that the transition from the solid to semisolid phase state of sucrose nanoparticles occurs at ∼18% RH as reported previously for sub-micrometer sucrose particles,^57^ the restructuring is likely more evident at and above 18% RH as a result of progressively lower viscosity of the semisolid particle.^57^ We note that, at elevated RH where sucrose nanoparticles become liquid, the nanoparticle restructuring effect on the measured GF should diminish, and as we demonstrate below, the GF measurements on sucrose nanoparticles at RH > 60% overlap well with the measurements on larger particles and theoretical predictions. The occurrence of restructuring is revealed from the observed decrease in *D*_area_ at 18% RH relative to 7% RH (Figure 1D). This is likely due to the propensity to attain the particle shape that minimizes the particle surface energy, which is in part governed by the interactions between the nanoparticle surface and underlying solid substrate. Because the substrate surface is hydrophobic, the RH increase results in hydration of nanoparticles and their interactions with the underlying hydrophobic surface result in restructuring, where such a substrate effect becomes more significant for nanosized particles. The extent of nanoparticle restructuring is likely dependent upon the type and size of particles, their viscoelastic properties, and type of substrate used. Unlike the sub-micrometer sucrose particles, the nanoparticles are expected to more readily undergo the restructuring as a result of a larger surface/volume ratio compared to the sub-micrometer particles.^63,75,76^ The restructuring phenomenon observed herein was also reported previously on soot, ammonium sulfate, non-deliquesced NaCl, and carbonaceous aerosol nanoparticles deposited on various surfaces, where the particle size was shown to decrease as RH increased.^61,63,73,74^Figure 1E shows normalized *D*_area_ (relative to 7% RH) as a function of RH, where smaller nanoparticles display progressively lower normalized *D*_area_ compared to the larger nanoparticles. This affirms the expectation that smaller nanoparticles tend to undergo the restructuring more readily on the surface. The result highlights the significant size-dependent influence of the underlying surface toward studying the water uptake of individual nanoparticles.

**Figure 2:**
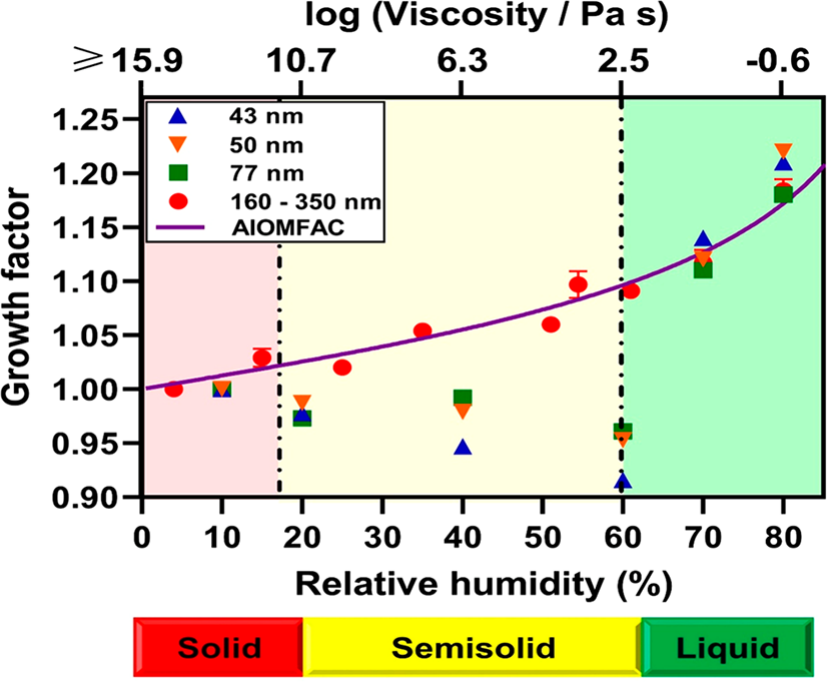
Plot of the AFM growth factor versus RH (bottom axis) or corresponding viscosity (top axis) for sucrose nanoparticles with heights (at 10% RH) of 43 nm (blue triangles), 50 nm (orange inverted triangles), and 77 nm (green squares) with corresponding *D*_vol_ of 206, 235, and 315 nm, respectively, and previous measurements on sub-micrometer sucrose particles with heights in the range of 160–350 nm and corresponding *D*_vol_ range of 400–1150 nm (red circles) as a reference.^57^ The RH range for the solid, semisolid, and liquid phase states is indicated by red, yellow, and green color bars, respectively. The RH–viscosity relationship is taken from Song et al. This was reproduced from ref (78). Copyright 2016 American Chemical Society (ACS). The purple line represents theoretical prediction of the growth factor using the AIOMFAC model from Hodas et al. This was reproduced from ref (77). Copyright 2015 Copernicus Publications.

To further explore the applicability of the AFM GF measurements on sub-100 nm particles, GF was measured on several sucrose nanoparticles over a wider RH range. Figure 2 shows the AFM GF versus RH for three selected individual sucrose nanoparticles [heights of 43 nm (blue triangles), 50 nm (orange inverted triangles), and 77 nm (green squares) with corresponding *D*_vol_ of 206, 235, and 315 nm, respectively, at 10% RH] over the ∼10–80% RH range. In addition, GF data measured on larger sucrose particles (height range of 160–350 nm and *D*_vol_ range of 400–1150 nm at 7% RH) from Lee et al.^57^ and theoretical prediction using the aerosol inorganic–organic mixtures functional groups activity coefficients (AIOMFAC) model from Hodas et al.^77^ are shown as a reference. For the RH range at and below 60%, each nanoparticle GF is decreasing with an increase in RH, where the extent of GF reduction is higher for smaller particles, consistent with the results shown in Figure 1. However, the GF values at RH greater than 60% start to overlap reasonably well with both the theoretical prediction and results obtained on sub-micrometer sucrose particles. Because sucrose particles at the 60–80% RH range are expected to be liquid, as shown below, these results suggest that, while the GF measurements on sub-100 nm semisolid nanoparticles could lead to inaccurate determination of the GF, such measurements become more accurate once particles are in the liquid phase. We note, however, that, despite the semisolid nanoparticle restructuring that results in lower than expected GF values at RH below 60%, the extent of actual water uptake and corresponding solute concentration, which can be inferred from the phase state measurements to be discussed next, is comparable to the sub-micrometer particles. The AFM-based contact mode force spectroscopy at various RH was next used to determine the phase state of sucrose nanoparticles as a function of RH and identify humidity values where transitions between the solid, semisolid, and liquid phase states occur.

**Figure 3:**
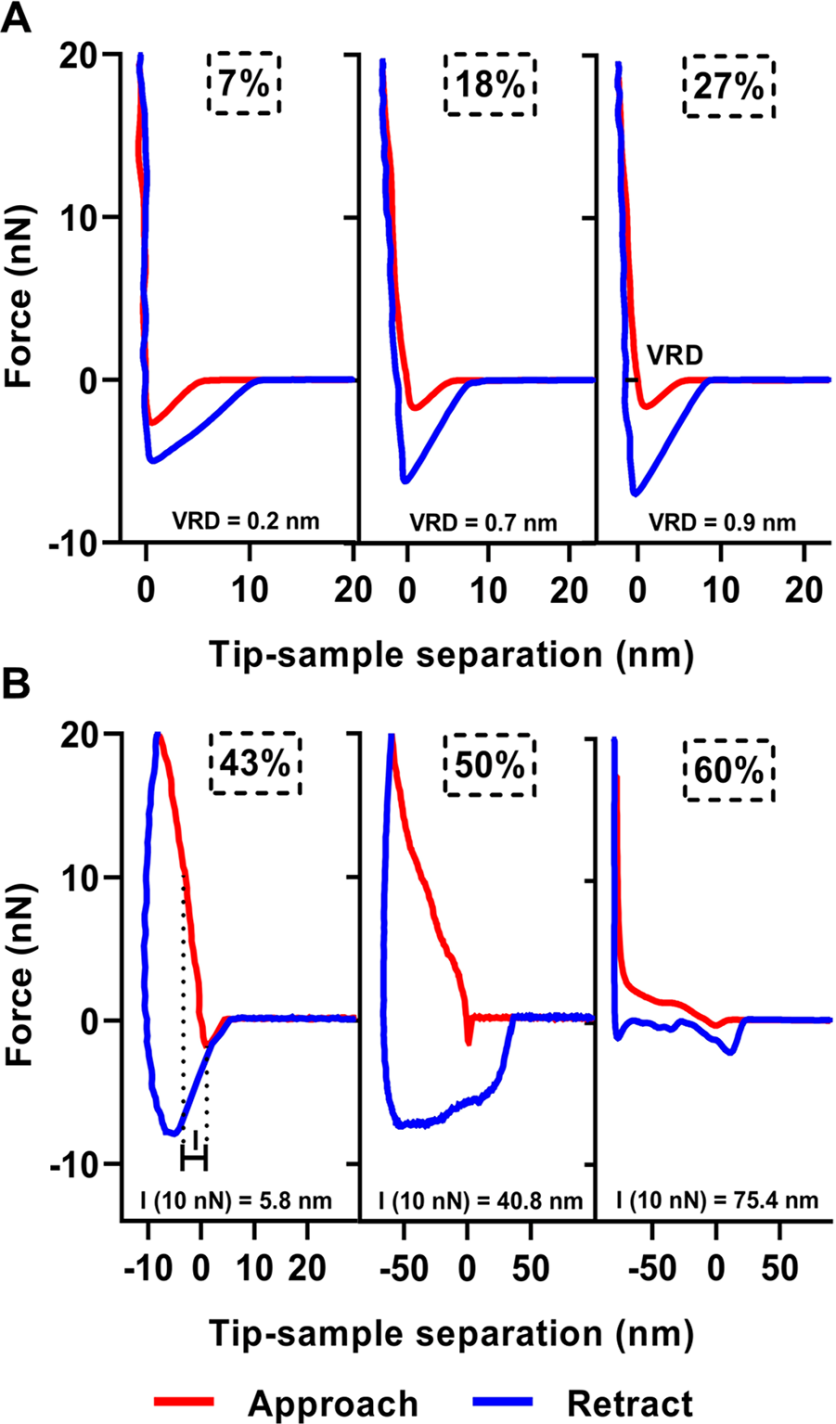
Representative force versus tip–sample separation plots for selected RH ranging from 7 to 60% for an individual sucrose nanoparticle (70 nm height and *D*_vol_ of 155 nm at 7% RH) with the maximum applied force of 20 nN. The approach and retract data for the AFM probe moving toward and away from the particle surface are shown in red and blue curves, respectively. (A) VRD measurement is shown along with the corresponding values measured for the force plots collected at 7, 18, and 27% RH. (B) *I* measurement at the applied force of 10 nN is shown along with the corresponding values for the force profiles collected at 43, 50, and 60% RH.

Figure 3 shows representative force versus tip–sample separation plots collected over an individual sucrose nanoparticle (70 nm in height and *D*_vol_ of 155 nm at 7% RH) at varying selected RH values ranging from 7 to 60%. Each force plot was collected at an approximate center of each particle. The force plots for the sucrose nanoparticle are qualitatively similar to those previously reported for sucrose sub-micrometer particles.^57^ For each force plot at a particular RH, the viscoelastic response distance (VRD) and indentation depth (*I*) at 10 nN were determined on the basis of the previously established method, as illustrated in Figure 3.^57,65^ The VRD values can be related to the particle viscoelastic nature, where higher values generally correspond to lower viscosity. The relative indentation depth (RID) at 10 nN was quantified by dividing the measured indentation depth at 10 nN by the particle height at the corresponding RH. Previously, a quantitative framework was established to determine the phase state of individual sub-micrometer particles using the VRD and RID measurements.^65^ Specifically, the RID measurement is used to differentiate between the semisolid and liquid phases, where a RID value equal or greater than 0.95 is indicative of a liquid and a value less than 0.95 is indicative of a semisolid or solid phase. The VRD measurement is used to differentiate between the solid and semisolid phases, where a VRD value less than 0.5 nm is indicative of a solid phase and a value greater than 0.5 nm is indicative of a semisolid phase.

**Figure 4:**
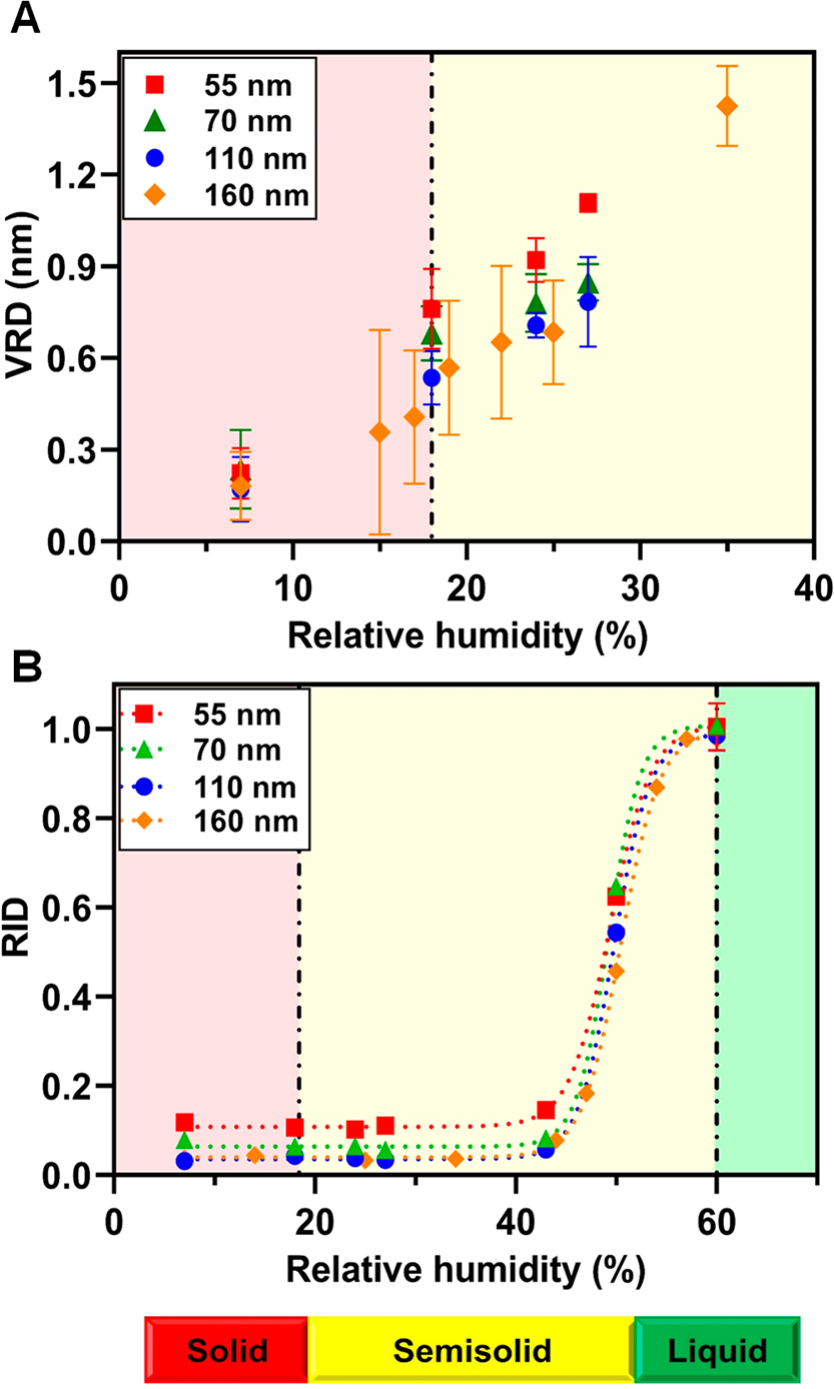
AFM (A) VRD and (B) RID measured at 10 nN versus RH collected over three individual sucrose nanoparticles with the heights (at 7% RH) of 55, 70, and 110 nm and *D*_vol_ of 130, 155, and 230 nm, respectively. The RID and VRD data for an individual sucrose particle with height (at 7% RH) of 160 nm and *D*_vol_ of 400 nm are plotted as a reference. This figure was reproduced from ref (57). Copyright 2017 American Chemical Society (ACS). The error bars represent one standard deviation for each data set. The dotted lines represent the fit using a four-parameter logistic sigmoidal function and are for illustrative purposes only. The expected RH values for solid to semisolid and semisolid to liquid phase transitions are shown by the dash-dotted vertical black lines. The RH ranges for the solid, semisolid, and liquid phase states of the sucrose nanoparticles are indicated by the red, yellow, and green color bars, respectively.

Figure 4 shows VRD and RID measurements with respect to RH over three selected sucrose nanoparticles (particle heights of 55, 70, and 110 nm with *D*_vol_ of 130, 155, and 230 nm, respectively, at 7% RH) along with the previously reported data for a single sub-micrometer sucrose particle (height of 160 nm and *D*_vol_ of 400 nm at 7% RH). All particles display VRD values less than 0.5 nm at 7% RH, and the VRD values become greater than 0.5 nm at 18% RH, indicative of the phase transition between the solid and semisolid phase states that occurs between these two RH values.^57^ The RID values at 10 nN for all particles are lower than 1 below 60% RH and become equal to 1 at 60% RH, indicative of the semisolid to liquid phase transition.^57^ Over the RH range below 43%, the RID values were not changing significantly, which is expected for a relatively stiff particle in the solid and semisolid phase state that results in relatively low indentation depths of 4–6 nm. However, as the RH increases from 43 to 60%, as a result of significant lowering of the particle viscosity during water uptake, a significant increase in the indentation depth occurs from ∼6 to 75 nm, resulting in a RID value of 1 at 60%, which is indicative of the particle in the liquid phase.^57^ Noteworthy, as the particle height decreases from 160 to 50 nm, a systematic increase in the RID values measured at RH below 60% was observed. Because the indentation depths at a particular RH below 60% were comparable for all nanoparticles with different heights studied here, the lower nanoparticle height contributes to a larger corresponding RID value. Despite this, however, the RID measurements and semisolid to liquid phase transition identification are applicable, because the RID values are only evaluated near the 0.95–1 range to identify the phase transition. The RH values where solid to semisolid and semisolid to liquid phase transitions occurred were ∼18 and 60% RH, which, for sucrose, are equivalent to viscosities of 10^11.2^ and 10^2.5^ Pa s, respectively, based on the viscosity measurements performed on sub-micrometer particles.^57,78^ Overall, both the VRD and RID results over individual sucrose nanoparticles show that the phase state methodology established previously for sub-micrometer particles can be similarly extended to nanoparticles with heights as low as 50 nm and volume equivalent diameters as low as 100 nm. As a result of a close overlap of RH values, where each phase transition is expected to occur, we can conclude that limitations of the AFM rather than a significant difference in water uptake yielded lower GF values for sucrose nanoparticles relative to the sub-micrometer counterparts.

**Figure 5:**
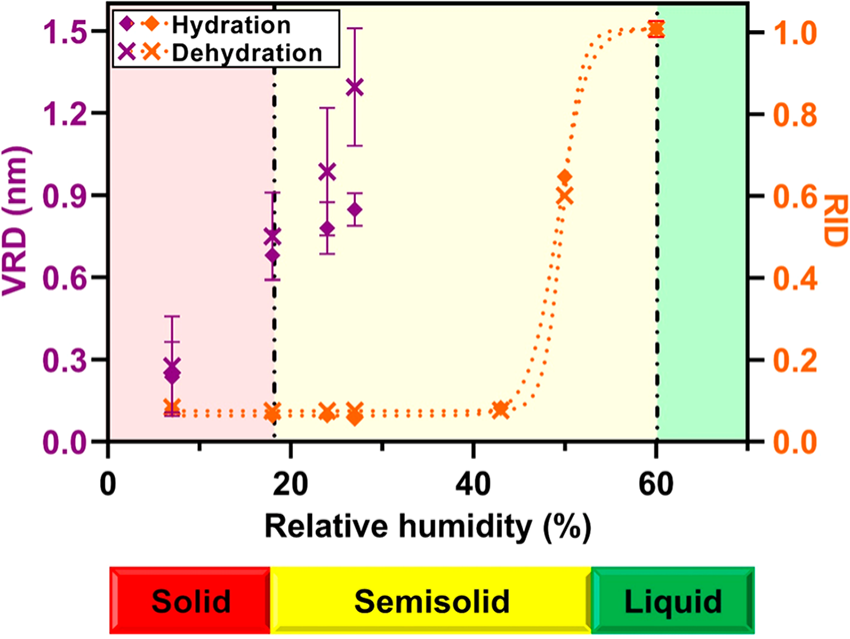
AFM (left) VRD and (right) RID at 10 nN versus RH collected over an individual 70 nm in height and *D*_vol_ of 155 nm (at 7% RH) sucrose nanoparticle during the hydration (diamonds) and dehydration (crosses) cycle. The error bars represent one standard deviation for each data set. The dotted lines represent the fit using a four-parameter logistic sigmoidal function and are for illustrative purposes only. The expected RH values for solid to semisolid and semisolid to liquid phase transitions are shown by the dash-dotted vertical black lines. The RH ranges for the solid, semisolid, and liquid phase states of the sucrose nanoparticles are indicated by the red, yellow, and green color bars, respectively.

To further validate the nanoparticle phase state measurements, the VRD and RID values were also measured at various RH during both the hydration and dehydration modes for an individual 70 nm in height and *D*_vol_ of 155 nm (at 7% RH) sucrose nanoparticle (Figure 5). Both the VRD and RID data show reasonably close overlap between the hydration and dehydration measurements and yield expected phase transition RH values of ∼18 and 60% for the solid to semisolid and semisolid to liquid phase transitions, respectively.^57^ Note, somewhat higher VRD values observed at RH > 20% during the dehydration mode relative to the hydration mode are likely due to the presence of an additional amount of water at the surface of the particle and AFM probe. However, despite such small deviation, the solid to semisolid phase state determination method based on the VRD measurements is applicable and accurate for either the hydration or dehydration modes.

## Conclusion

In summary, our findings establish the AFM force spectroscopy as an accurate method to determine the phase state of individual nanoparticles over a wide range of RH. The water uptake studies of substrate-deposited individual sucrose nanoparticles showed that, as RH increased up to 60%, the particle height increased with the concurrent decrease in the projected area diameter, which collectively resulted in the overall decrease of the GF. The decreasing GF with increasing RH up to 60% could be attributed to the substrate effects that result in the semisolid nanoparticle restructuring. At RH > 60%, sucrose nanoparticles are in the liquid phase and quantified GFs overlap well with the sub-micrometer particles and theoretical predictions. This suggests that quantification of the GF of nanoparticles may be inaccurate over the RH range where particles are semisolid but becomes accurate at elevated RH where particles are liquid. Despite this, however, application of the AFM phase state method on individual sucrose nanoparticles (particle heights as low as 50 nm and volume equivalent diameter of 100 nm) revealed a close overlap in the solid–semisolid and semisolid–liquid phase transitions between the sub-micrometer and sub-100 nm sucrose particles. Thus, despite the nanoparticle restructuring, the extent of water uptake and corresponding nanoparticle viscosity at a particular RH is comparable to the sub-micrometer particles. Furthermore, the phase determination method was shown to be applicable and accurate for either the hydration or dehydration modes. This AFM methodology enables direct determination of the morphology, size, and phase state of individual sub-100 nm aerosols as a function of RH that could enable a better understanding on how the particle-to-particle variability of the phase state of aerosols influences atmospheric processes.

## Notes

The data for this publication can be retrieved from the University of California, San Diego Library Digital Collections, 10.6075/J0S46QG6.
